# Physiological and Pathophysiological Roles of Metabolic Pathways for NET Formation and Other Neutrophil Functions

**DOI:** 10.3389/fimmu.2022.826515

**Published:** 2022-02-09

**Authors:** Darko Stojkov, Lea Gigon, Shuang Peng, Robert Lukowski, Peter Ruth, Alexander Karaulov, Albert Rizvanov, Nickolai A. Barlev, Shida Yousefi, Hans-Uwe Simon

**Affiliations:** ^1^ Institute of Pharmacology, University of Bern, Bern, Switzerland; ^2^ Department of Pharmacology, Toxicology and Clinical Pharmacy, Institute of Pharmacy, University of Tübingen, Tübingen, Germany; ^3^ Department of Clinical Immunology and Allergology, Sechenov University, Moscow, Russia; ^4^ Institute of Fundamental Medicine and Biology, Kazan Federal University, Kazan, Russia; ^5^ Institute of Cytology, Russian Academy of Sciences, St. Petersburg, Russia; ^6^ Regulation of Cell Signaling Laboratory, Moscow Institute of Physics and Technology, Dolgoprudny, Russia; ^7^ Institute of Biochemistry, Brandenburg Medical School, Neuruppin, Germany

**Keywords:** neutrophil, metabolism, neutrophil extracellular traps, metabolic switch, glycolysis

## Abstract

Neutrophils are the most numerous cells in the leukocyte population and essential for innate immunity. To limit their effector functions, neutrophils are able to modulate glycolysis and other cellular metabolic pathways. These metabolic pathways are essential not only for energy usage, but also for specialized effector actions, such as the production of reactive oxygen species (ROS), chemotaxis, phagocytosis, degranulation, and the formation of neutrophil extracellular traps (NETs). It has been demonstrated that activated viable neutrophils can produce NETs, which consists of a DNA scaffold able to bind granule proteins and microorganisms. The formation of NETs requires the availability of increased amounts of adenosine triphosphate (ATP) as it is an active cellular and therefore energy-dependent process. In this article, we discuss the glycolytic and other metabolic routes in association with neutrophil functions focusing on their role for building up NETs in the extracellular space. A better understanding of the requirements of metabolic pathways for neutrophil functions may lead to the discovery of molecular targets suitable to develop novel anti-infectious and/or anti-inflammatory drugs.

## Introduction

Neutrophils are the most abundant white blood cells in the human peripheral blood with an estimated daily turnover of 10^10^ to 10^11^ cells (1, 2). They exhibit a characteristic nucleus with 3 to 5 segmented lobes which are connected to a thin strip of nuclear material (3). Neutrophils are generated in the bone marrow in a regulated process of granulopoiesis. They are terminally differentiated cells with a short half-life time under physiological conditions (4–6). Therefore, they need to be constantly replenished from bone marrow precursor cells (3). The key role of neutrophils is to respond and diminish extracellular pathogens and to participate in the initiation of the adaptive immune response (2, 5). In addition, neutrophils perform diverse cellular functions, including oxidative burst, phagocytosis, degranulation, production of different cytokines and chemokines, and neutrophil extracellular trap (NET) formation (2, 7). Many neutrophil functions depend on energy and cytoskeleton reorganization (8, 9).

Neutrophils are equipped with a specialized enzyme system, so-called nicotinamide adenine dinucleotide phosphate (NADPH) oxidase (often referred to as NOX2), which is able to produce reactive oxygen species (ROS) in higher quantity as compared to ROS generated by mitochondria. The NADPH oxidase reduces molecular oxygen to superoxide, that serves as a precursor for formation of hydrogen peroxide contributing to bacterial killing in order to dampen the inflammatory response (10). During their anti-microbial activity, neutrophils release granule proteins (3). Three main types of granules can be distinguished in neutrophils. During the promyelocyte stage the azurophilic granules are formed. These primary granules are large and dense, and contain myeloperoxidase (MPO) (11). Lactoferrin is found in specific or secondary granules, which develop during the myelocyte-metamyelocyte phase (12). At the band-stage of neutrophil development, the tertiary granules are formed that contain gelatinase (13). Neutrophils release many cytokines, including interleukin (IL)-6, IL-1, and tumor necrosis factor alpha (TNF-α), which aid in the inflammatory process by attracting additional white blood cells to the inflammation site (14, 15).

When activated, neutrophils migrate through chemotaxis towards the inflammatory sites (16) to combat the invading microorganisms. The precise mechanisms of anti-microbial inflammatory responses of neutrophils are still under intense investigations. Neutrophils are capable of killing microorganisms both intracellularly by phagocytosis and extracellularly by forming NETs containing released DNA and granule proteins (17–19). We and others have reported that the released web-like structure is composed of mitochondrial DNA (mtDNA) and granule proteins (8, 9, 19–26). NETs ensure the elimination of pathogens by preventing their spread and increase the local concentration of antimicrobial and toxic factors (27). Eosinophils, basophils, mast cells, and monocytes/macrophages can also form extracellular DNA traps (28–35).

Since the discovery of NETs, several microbial and noninfectious stimuli have been identified to activate neutrophils to induce NET formation (24, 36–39).

The production of ROS seems to be essential for NET formation (19, 40–42). Increased intracellular ROS activates actin and tubulin glutathionylation, tightly regulated by glutaredoxin 1 (Grx1), an enzyme required for deglutathionylation of actin and microtubules. Hence, an active cytoskeleton movement is vital for the formation of NETs (8). Moreover, the re-organization of the microtubule arrangements and consequently NET formation have been shown to depend on glycolytic ATP (9). To execute effector functions, neutrophils rely on simple metabolic pathways, such as glycolysis (43). Despite the fact that glycolysis remains the major metabolic pathway in the cytosol of neutrophils, more recent studies have identified additional metabolic routes in these cells (3). For instance, neutrophils utilize the pentose-phosphate pathway (PPP) to produce NADPH and ribose. In certain conditions, mainly due to limited availability of glucose, neutrophils are also able to adapt to fatty acid metabolism and glutaminolysis. On the contrary, when glucose is abundant, they can store small amounts of glycogen (44). In this review, we discuss the metabolic pathways used by neutrophils and explore the metabolic changes during NET formation.

## Neutrophil Functions and Associated Metabolic Pathways

In the last decade, there has been a great interest to investigate the impact of metabolic activity on regulatory pathways in immune cells, including neutrophils (45–48). Compared to other immune cells such as macrophages and lymphocytes, neutrophils have modest numbers of mitochondria and they obtain their energy mainly from glycolysis (49, 50). Although initial analyses using electron microscopy suggested a few functional mitochondria in mature neutrophils only (51, 52), recently developed mitochondrial probes and new methods to quantify both oxygen consumption rate (OCR) and extracellular acidification rate (ECAR) resulted in novel insights regarding the functional role of mitochondria in neutrophils (3, 9). It is now well established that neutrophils express components of the oxidative phosphorylation (OXPHOS) complex (50) and exhibit an active mitochondrial network (53). They exhibit functional mitochondria (54) and changes in mitochondrial potential (**Δ**Ψm) were detected in activated mature neutrophils (50, 53). It has also been reported that the optic atrophy 1 (OPA1) protein, known to be responsible for controlling mitochondrial fusion and maintaining the mitochondrial cristae junctions tight to allow efficient oxidative respiration (55–57), is also required for ATP production through glycolysis in neutrophils (9). Moreover, functional mitochondria and glycolytic ATP production were necessary to form an intact microtubule network and functional NETs, both contributing to the anti-microbial defense mediated by neutrophils in an *in vivo* model of *Pseudomonas aeruginosa* (*P. aeruginosa*) lung infection (9).

**Figure 1:**
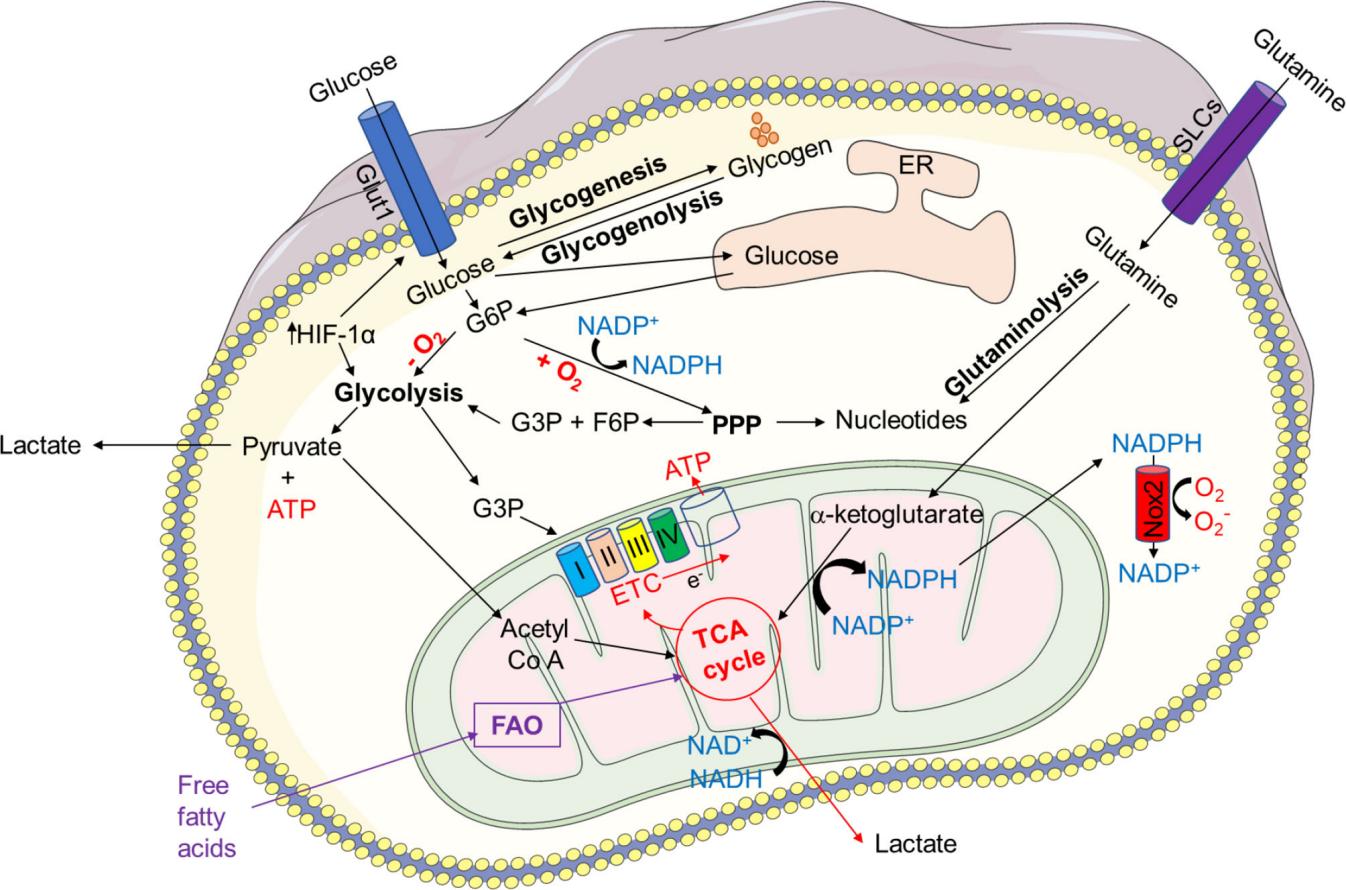
Overview of key metabolic pathways in neutrophils. In neutrophil cytosol, glycolysis is a primary metabolic route that transforms glucose to pyruvate via a number of enzymes and reactions. Pyruvate is converted to lactate and released out of the cells during anaerobic glycolysis in absence of oxygen. Pyruvate contributes to the TCA cycle after conversion to acetyl-coenzyme A (acetyl Co A) in the presence of oxygen, which results in reducing energy intermediates NADH and dihydroflavine-adenine dinucleotide (FADH2) to generate ATP via the electron transport chain (ETC). Neutrophils also use the pentose-phosphate pathway (PPP) to generate NADPH and riboses, which are then used to build nucleotides, by employing glucose-6-phosphate, a glycolytic pathway intermediate, as an entry point during the oxidative and non-oxidative phases to manufacture NADPH and riboses. NADPH modulates redox signaling and is required for NADPH oxidase-dependent ROS production in neutrophils. Glycogen reserves become concentrated in neutrophils when glucose levels rise, providing glucose-based glycolytic intermediate supply on demand. Through fatty acid synthesis (FAS), the TCA cycle intermediate citrate can be converted to free fatty acids, which can then be transported from the extracellular environment through the FAO pathway to produce acetyl-CoA, which fuels the TCA cycle and produces significantly more energy in the form of ATP. Glutamine helps the TCA cycle by producing α-ketoglutarate through glutaminolysis. Neutrophils also use the glycerol-3-phosphate shuttle to create NAD^+^ from NADH, which aids in mitochondrial membrane potential. HIF-1α augments the activity and expression of GLUT1 and GLUT3, resulting in increased glucose uptake, elevated hexokinase 2 (HK2) and phosphofructokinase B3 (PFKFB3) enzymatic activities, and enhanced ATP production.

Metabolic activities in neutrophils are not yet completely understood, and several metabolic pathways, including glycolysis, *via* tricarboxylic acid (TCA) cycle (also known as Krebs cycle), pentose phosphate pathway (PPP) *via* OXPHOS, glycogenolysis, glutaminolysis, and fatty acid β-oxidation (FAO) are considered to be utilized by neutrophils to fulfill the energetic, biosynthetic, and functional requirements of neutrophils (3, 43, 58–62) (**Figure 1**).

Even though glycolysis is a dominant metabolic pathway for neutrophils, under glucose depletion, neutrophils mostly rely on the breakdown of stored glycogen (glycogenolysis) (63, 64), and certain functions of neutrophils, such as phagocytosis, require stored glycogen (65). The PPP or hexose monophosphate (HMP) shunt, which uses the glycolytic intermediate glucose-6-phosphate (G6P) to create ribose-5-phosphate (R5P) and NADPH in the cytosol, is required for the formation of ROS by the NADPH oxidase in neutrophils (58). Furthermore, in case of limited glucose availability, neutrophils activate a compensatory metabolism of fatty acids (66). Mitochondrial FAO transforms fatty acids to acyl-CoAs, which then enters the TCA cycle as acetyl-CoA, and energy in form of ATP is generated through the electron transport chain (3, 47, 67). Glutamine is another metabolic substrate used by neutrophils, especially when glucose levels are low (3, 68–70). Glutamate dehydrogenase produces α-ketoglutarate, which feeds the TCA cycle (70).

Neutrophils are frequently found in microenvironments with low oxygen levels. To survive under hypoxia condition, neutrophils retain hypoxia-inducible factor-1α (HIF-1α) and factor inhibiting HIF (FIH) hydroxylase oxygen-sensing pathway to diminish neutrophil apoptosis (71, 72). It has been described that hypoxia inhibits apoptosis by inducing a number of glycolytic enzymes, including GAPDH, allowing for the ongoing production of ATP, which is critical for neutrophil survival and function (73). Under hypoxic conditions, HIF-1α knockout mouse neutrophils are more prone to apoptosis (71). Moreover, HIF-1α and mammalian target of rapamycin (mTOR) have been also identified as important regulators of glycolysis in neutrophils (74, 75). HIF-1α upregulates the enzymes hexokinase 2 (HK2) and phosphofructokinase B3 (PFKFB3) to enhance the activity and expression of glucose transporter (GLUT) 1 and GLUT3 on neutrophils, resulting in increased glucose absorption, glucose metabolism, and ATP generation (74, 76, 77). HIF-1α has also been implicated in NET formation (78, 79). Increased NET formation has been associated with HIF-1α stabilization. Thus, reduced NET formation and NET-mediated extracellular bacteria killing have been described in case of pharmacologic or genetic knockdown of HIF-1α (80). Additionally, HIF-2α, which has overlapping activities with HIF-1α, is also expressed by neutrophils, and its expression has been up-regulated under inflammatory circumstances (81).

Taken together, metabolic flexibility under a variety of stress conditions aids in meeting energy demands by efficiently diverting intermediate metabolites created by the operating metabolic pathways (82). In **Figure 1**, we describe major metabolic pathways, which supply the required energy for diverse neutrophil functions. In addition, we summarize the recent publications reporting defect in NET formation under pathological conditions where the metabolic activities of neutrophils are compromised (**Table 1**).

**Table 1:** Metabolic changes in NET-associated diseases.

Diseases	Metabolic shift	NET formation	Outcome	References
Diabetes mellitus	Decreased PPP Increased glycolysis	Increased **↑**	Increased diabetic ocular diseases Impaired diabetic wound healing Inflammation and tissue damage	(66, 83–88)
Obesity	Increased PPP Increased glycolysis	Increased **↑**	Increase of thromboembolic incidents High risk of cardiovascular occurrences	(89, 90)
Cancer	Increased glycolysis Increased FAO	Increased **↑**	Promotes breast cancer and liver metastasis Increased tumor growth	(26, 91–95)
SLE	Decreased glycolysis	Increased **↑**	High degree of inflammation and tissue destruction, endothelial damage Autoimmunity and type I IFN signatures	(3, 24, 96–102)
RA	Increased glycolysis	Increased **↑**	Cartilage damage Immune activation	(102–106)
G6PDD	Decreased PPP	Decreased **↓**	Impaired microbicidal and metabolic activity Susceptibility to infection	(58, 107–109)
Sepsis	Increased glycolysis Increased PPP Increased FAO	Increased **↑**	Enhanced sepsis severity and organ damage Intestinal barrier dysfunction by promoting inflammation and epithelial apoptosis	(63, 110–119)
CF	Increased glycolysis	Increased **↑**	Enhanced inflammation Defective clearance of infections Decreased neutrophil apoptosis	(120–124)
COVID-19	Increased glycolysis	Increased **↑**	Enhanced inflammation Increased organ damage and mortality	(124–129)
Atherosclerosis	Increased FAO	Increased **↑**	Enhanced inflammation and endothelial cell damage	(130–132)

### Glycolysis

Glycolysis is a key metabolic pathway that converts glucose to pyruvate to generate small quantities of ATP and NADPH (3, 49, 50, 133, 134). Glycolysis is the foremost metabolic pathway in most immune cells, including neutrophils, which depend on glycolysis as a source of ATP (43). This fundamental metabolic pathway does not require oxygen and represents a sequence of ten reactions catalyzed by enzymes (43, 65). During glycolysis, neutrophils uptake extracellular glucose through glucose transporters (GLUTs), and express various GLUTs, such as GLUT1, GLUT3, and GLUT4 (135). Upon activation, increased surface expression of GLUTs is observed followed by an increase in the uptake of glucose. Once inside the cell, glucose is immediately converted into glucose-6-phosphate by the hexokinase enzymes (136). Each G6P molecule is converted into two molecules of pyruvate and two molecules of ATP as well as NADH. In the presence of oxygen (aerobic conditions), pyruvate can be used to make acetyl-Co A, which can then be oxidized *via* the TCA cycle to produce energy (ATP through ETC) (3, 47, 67, 134). Many studies have demonstrated the essential role of glucose in neutrophils, whereby the depletion of glucose completely terminated most of neutrophil effector functions (43, 49, 58, 63) (**Figure 1** **)**

### Pentose-Phosphate Pathway (PPP)

An alternative glucose-dependent metabolic pathway in neutrophils is the PPP, also known as the hexose monophosphate shunt, which has been seen in both resting and activated neutrophils as well as during NET formation (58). PPP is involved in the NADPH oxidase (NOX)-dependent ROS production and consequently contribute to NET formation (58). Superoxide production *via* the PPP is an essential catalysator in the process of bacteria killing by neutrophils (137). Defects in the PPP pathway or inhibition of the PPP key enzyme, glucose-6-phosphate dehydrogenase (G6PD), under high glucose concentrations led to reduced ROS production (138).

**Figure 2:**
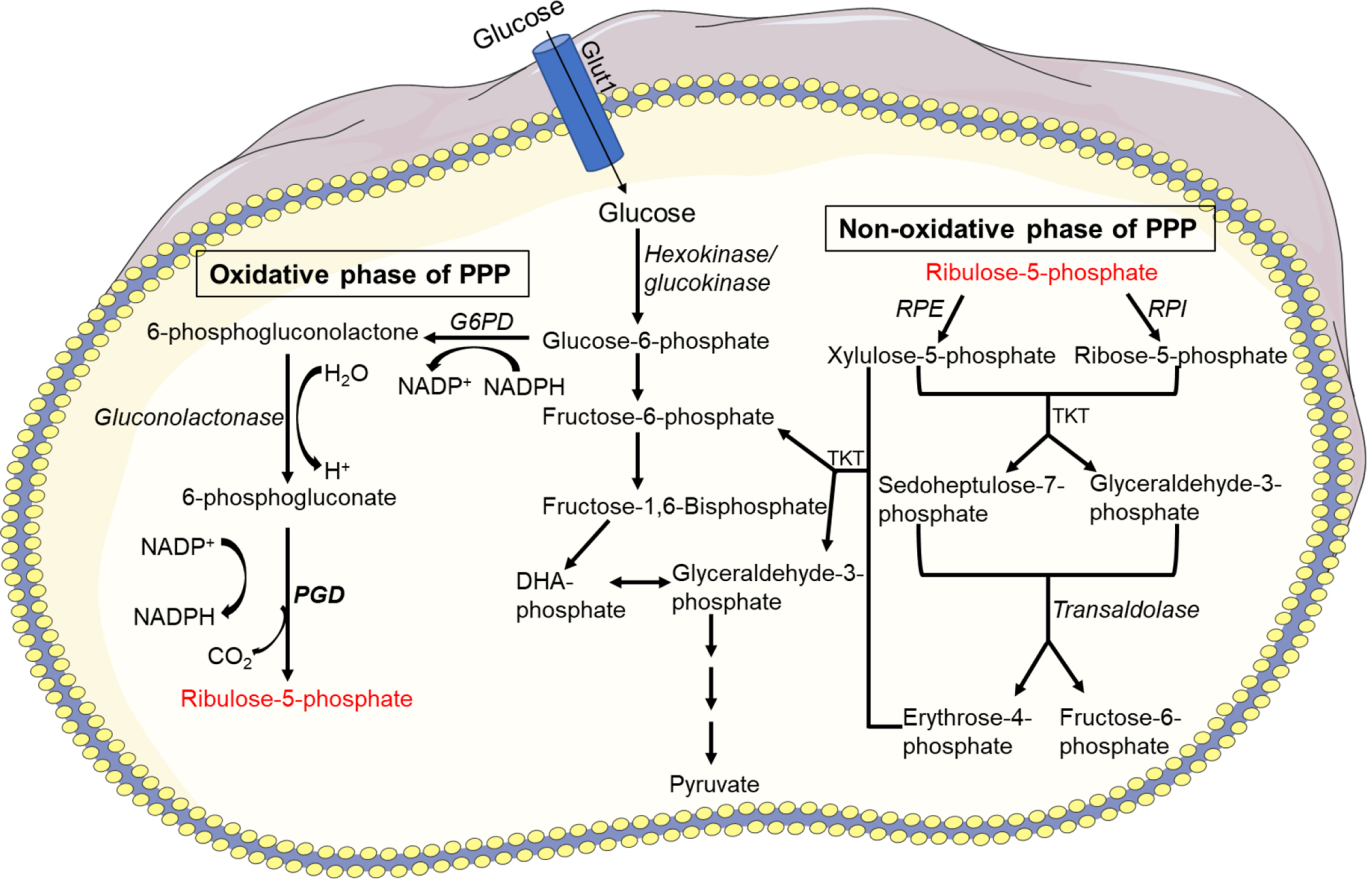
Pentose phosphate pathway (PPP). There are two phases in the PPP: oxidative and non-oxidative. G6P-dehydrogenase (G6PD), 6-phosphogluconolactonase, and 6-phosphogluconate dehydrogenase (PGD) convert G6P into CO_2_, ribulose-5-phosphate, and NADPH during the oxidative phase to maintain redox balance under stressful conditions. In the non-oxidative phase, enzymes such as ribose-5-phosphate isomerase, ribulose-5-phosphate 3-epimerase, a transketolase, and a transaldolase convert ribulose-5-phosphate to nucleic acids, sugar phosphate precursors, or glycolytic precursors such as fructose-6-phosphate (F6P) and glyceraldehyde-3-phosphate (G3P). PPP and glycolysis share a pool of G3P and F6P, resulting in lactate or pyruvate production.

The PPP encloses an oxidative and a non-oxidative phase (**Figure 2**). During the oxidative phase, G6PD, 6-phosphogluconolactonase, and 6-phosphogluconate dehydrogenase (PGD) convert G6P into CO_2_, ribulose-5-phosphate, and NADPH, to maintain redox equilibrium during cell stress. Several enzymes are involved in the non-oxidative phase, including ribose-5-phosphate isomerase, ribulose-5-phosphate 3-epimerase, a transketolase (TKT), and a transaldolase (TAL), which convert ribulose-5-phosphate to nucleic acids, sugar phosphate precursors, or glycolytic precursors like fructose-6-phosphate (F6P) and glyceraldehyde-3-phosphat (G3P). PPP and glycolysis share a pool of G3P and F6P, which results in lactate or pyruvate production, respectively.

### Glycogen Metabolism

Glycogen, a multibranched polysaccharide stored in the neutrophil cytosol, is the central storage of glucose, and glycogenolysis, a breakdown of glycogen to glucose-1-phosphate, is a signal of limited availability of glucose and ATP (63, 139). Neutrophils appear to be the only myeloid cell type capable of gluconeogenesis and glycogenesis, which reveals their capacity to create ATP under low-glucose and low-oxygen conditions (140). Glucose-1-phosphate is converted to glucose-6-phosphate, which is often hydrolyzed to glucose using glucose-6-phosphatases (G6Pase) *via* gluconeogenesis (141). In the absence of glucose, neutrophils rely on glycogenolysis to break down stored glycogen as a source of glucose (64). Neutrophils isolated from inflammatory sites accumulate more glycogen than neutrophils from the peripheral circulation (64, 139). As a result, during inflammation, glycolysis appears to be a prominent and the critical metabolic route for neutrophils. Glycogen content raises as neutrophils mature (142). Once sufficient glucose is available, and normal intracellular levels of glucose are re-established, synthesis of glycogen is resumed (49, 143).

**Figure 3:**
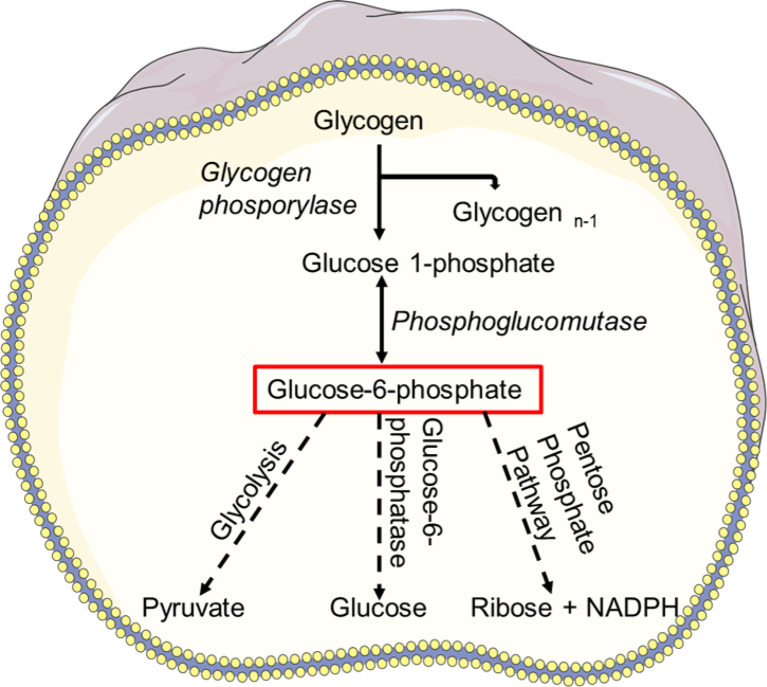
Glycogen metabolism. Breakdown of glycogen results in glucose-1 phosphate which is then converted to glucose-6-phosphate (G6P) by phosphoglucomutase. G6P can enter the glycolysis pathway and used as a source of energy. Using glucose-6-phosphatase (G6Pase), G6P can be hydrolyzed to glucose via gluconeogenesis and subsequently released into the blood. Glucose-6-phosphate can also be taken by PPP and converted to NADPH or ribose in a variety of tissues.

The interplay of glucose between endoplasmic reticulum (ER) and cytoplasm regulates the neutrophil glycogen level (**Figure 3**). Glucose cycling in the ER plays an essential role in neutrophil apoptosis and other neutrophil functions (141). Moreover, it has been reported that deficiency of the ER glucose-6-phosphatase-β (G6Pase-β also known as G6PC3) underlies the G6PC3–deficient congenital neutropenia syndrome. Neutrophils from these patients exhibited increased ER stress and apoptosis. G6PC3 converts G6P into glucose and phosphate, which enters the ER *via* the G6P transporter (G6PT). G6PC3 deficiency in these patients leads to reduced cytoplasmic concentrations of glucose, G6P, lactate, and ATP, leading to increased apoptosis and typical neutropenia (144). Increased enzymatic activity of glycogen phosphorylase leads to further accumulation of intracellular G6P which can be utilized in different metabolic pathways in neutrophils (145). Neutrophils are susceptible to fluctuations of intracellular and extracellular glucose levels or impaired glucose absorption and respond accordingly (146). Deficiency of glucose-6-phosphate translocase (G6PT/SLC37A4) leads to glycogen storage disease type Iβ (GSD-1β) (147). GSD-1β−deficient neutrophils accumulate glycolysis-inhibiting analog of glucose in the cytoplasm that affect protein glycosylation (148), ATP generation, NADPH synthesis. Subsequently, neutrophils of glycogen storage disease patients exhibit impaired chemotaxis, oxidative burst and bactericidal activity (147, 149, 150), and increased apoptosis (151). Interestingly, a recent study discovered that treating GSD-1β patients with a sodium glucose cotransporter 2 (SGLT2) inhibitor, an anti-diabetic drug inhibits renal reabsorption of glucose while facilitating excretion of the inhibitory glucose analog, resolving the neutropenia and neutrophil dysfunctions (152). These studies indicate the importance of glucose in energy metabolism and NADPH oxidase-dependent neutrophil functional responses, hinting the essential need to better understand the glucose flux in neutrophils (**Figure 3**).

### Glutamine Metabolism

**Figure 4:**
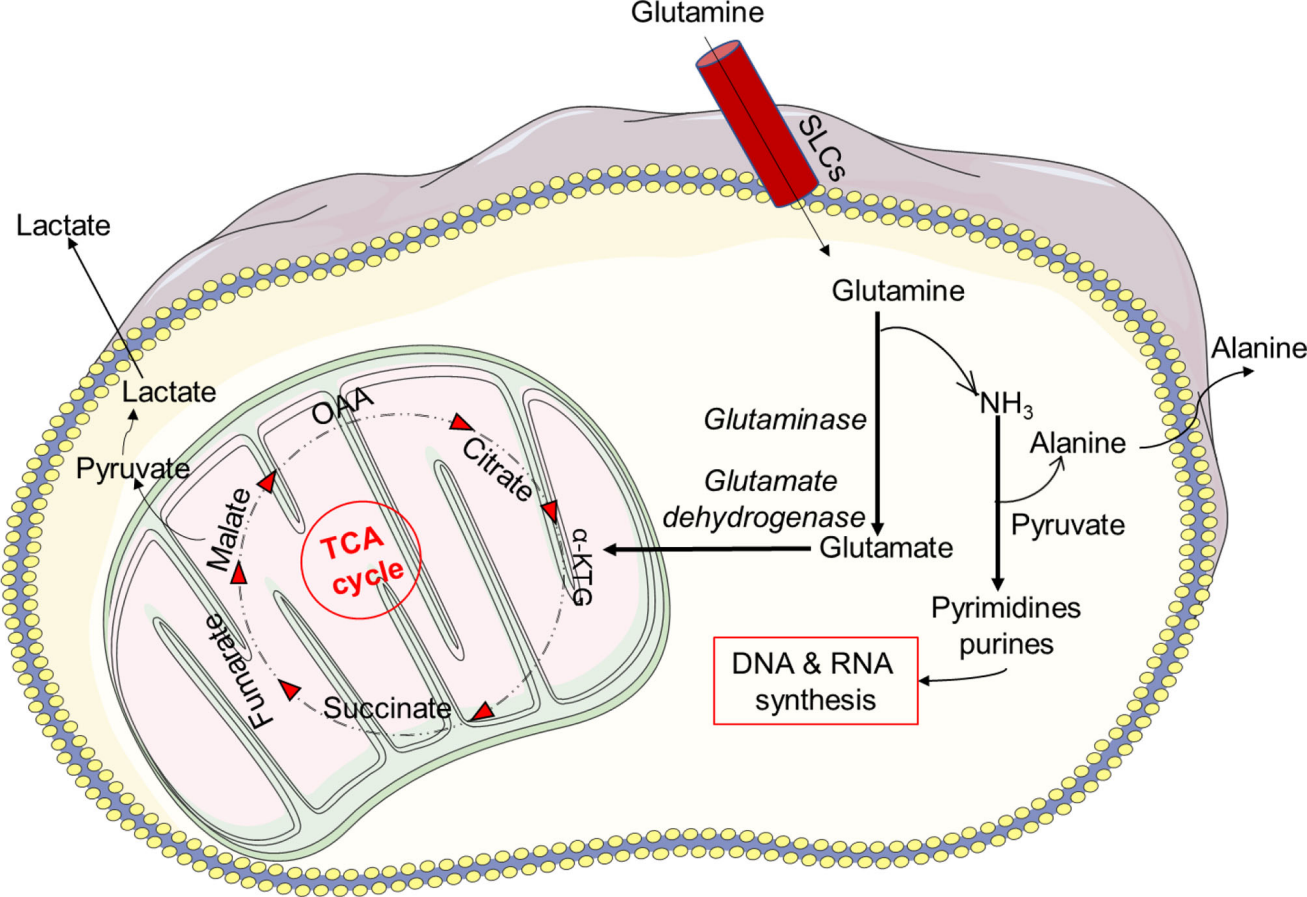
Glutamine metabolism. Glutamine metabolism results in glutamate, aspartate, lactate and ammonia production. Under physiological conditions glutamine is required for the synthesis of nucleotide precursors, such as RNA and DNA. The sodium-coupled neutral amino acid transporter (SNAT) family of proteins is one of several carrier type transporters (SLCs) responsible for bringing glutamine into the cell. Once inside the cell, glutamine is oxidized and transformed into glutamate. Furthermore, glutamate enters the mitochondria and becomes oxidized and metabolized to α-ketoglutarate, which allows NAD^+^ to be oxygenated and converted to NADH. The TCA cycle may accept α-ketoglutarate and create malate, which is subsequently transformed to pyruvate by the malate dehydrogenase, which oxygenates NADP^+^.

Glutamine is one of the most abundant amino acids in the body and can be used as a substrate in the biosynthesis of proteins, antioxidants, NADPH and other metabolic pathways involved in cellular integrity and cell functions (153). In a series of biochemical reactions, glutamine metabolism results in glutamate, aspartate, lactate and ammonia production (134). Glutamine is required for the synthesis of nucleotide precursors, such as RNA and DNA, in physiological conditions. When glucose availability is restricted under pathophysiological settings, many cells, including neutrophils, can shift to glutaminolysis metabolism to meet their energy requirements (70, 154–156). Once it enters the cell, glutamine needs to be oxidized in order to be transformed into glutamate (**Figure 4**). Glutamate then enters the mitochondria and becomes oxidized and metabolized to α-ketoglutarate, which allows NAD^+^ to be oxygenated and converted to NADH. The TCA cycle may accept α-ketoglutarate and create malate, which is subsequently transformed to pyruvate by the malate dehydrogenase, which oxygenates NADP^+^.

Pyruvate is subsequently converted into lactate in the cytosol or used by the mitochondria for OXPHOS. NAD^+^ can be restored through the generation of lactate in conditions of inflammation and low oxygen. How a cytosolic NAD^+^ enters mitochondria is still not known, especially because mammalian mitochondria do not generate NAD^+^ and are thought to be impermeable to pyridine nucleotides (134, 157). However, it has recently been shown that cytosolic NAD^+^ or NADH may be delivered directly into mammalian mitochondria (134, 157). Neutrophils may also consume glutamine at a faster rate than glucose (68). In addition, glutaminolysis, like PPP, plays a key role in the formation of NADPH and the expression of the NOX complex (158). *In vitro* studies have indicated that glutamine enhances bacterial killing by neutrophils from postoperative patients. The addition of 2 mM extracellular glutamine to neutrophils attenuated the adrenaline-induced suppression of superoxide generation, resulting in enhanced NADPH producing activity *via* glutamine metabolism (159, 160).

### Fatty Acids

Fatty acids are energy substrates for tissues and cells, including leukocytes, and involved in the maintenance of glucose and fuel homeostasis (161). The metabolism of fatty acids has an important role in the functional activity of neutrophils (162). The fatty acid chain length can vary from 3 to 30 carbon atoms, and, depending on the number of carbon atoms, the fatty acids are characterized as short-, medium, and long-chain fatty acids. Short-chain amino acids contain less than six carbon atoms. Those with six to ten carbon atoms are medium-chain fatty acids and long-chain fatty acids exhibit over twelve carbon atoms. The fatty acids can be monounsaturated with one carbon - carbon bond or polyunsaturated with two or more double bonds. In addition, the fatty acids with no double bonds in the molecule are known as saturated fatty acids (144, 163). The metabolism of fatty acids is increased when cells are exposed to limited glucose conditions. Utilization and oxidation of fatty acids occur in neutrophils and are important for their activation (164). Moreover, during neutrophil differentiation and maturation, fatty acids enter into the FAO pathway to produce ATP through OXPHOS (165). It has also been shown that fatty acids impaired glucose uptake and glucose metabolism (166).

**Figure 5:**
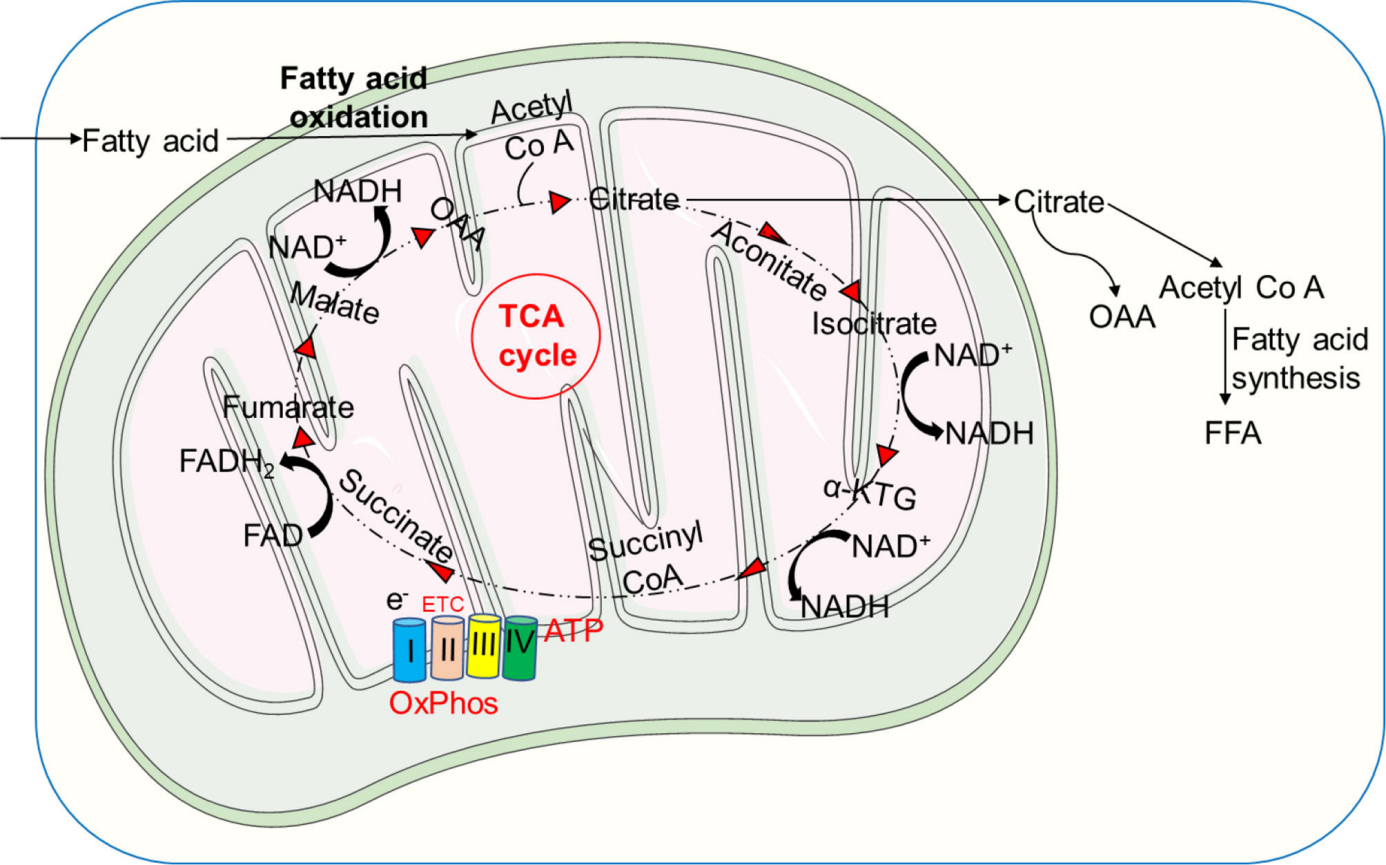
Metabolism of fatty acids. Fatty acids are oxidized to form acetyl-Co A, which is then converted to citrate by the activity of citrate synthase. Acetyl-Co A can be also produced by the mitochondrial matrix and further oxidized in the TCA cycle. Through FAS, the TCA cycle intermediate citrate can be converted to free fatty acids (FFA), which can then be transported from the extracellular environment via the FAO pathway to produce acetyl-Co A, fueling the TCA cycle to produce significantly more energy in the form of ATP.

Fatty acids are oxidized to produce acetyl-Co A, which is then converted to citrate *via* citrate synthase. The activation of pyruvate dehydrogenase kinase (PDK), which inactivates pyruvate dehydrogenase (PDH), is promoted by high acetyl-Co A/Co A and NADH/NAD ratios. ATP and citrate block phosphofructokinase (PFK), resulting in the buildup of G6P and inhibition of hexokinase, and therefore glycolysis is hindered. The mitochondrial matrix also produces acetyl-Co A, which can be oxidized in the TCA cycle (69) (**Figure 5**). Furthermore, carnitine, a quaternary ammonium compound, plays an important task in energy production due to its role in FAO by transporting long-chain fatty acids into mitochondria to be oxidized for energy production. Carnitine also participates in removing products of metabolism from cells. Additionally, acylcarnitines located in the outer mitochondrial membrane, are generated by carnitine palmitoyltransferase-1 (CPT-1) that catalyzes an ester bond of carnitine with long-chain fatty acids. Carnitine-acylcarnitine translocase (CACT) translocates acylcarnitines across the inner mitochondrial membrane (167). Acylcarnitines renew acyl-Co A inside the mitochondrion *via* the carnitine palmitoyltransferase-2 (CPT-2), which is located in the inner mitochondrial membrane (168). Carnitine returns to the cytoplasm for another cycle, whereas acyl-Co A enters the β-oxidation pathway, creating acetyl-Co A (under aerobic circumstances and low ATP levels) (167). The utilization of fatty acids by neutrophils and consequently the outcome of the interaction depends on different aspects, including the presence and absence of inflammation and the concentration of the fatty acids. Under *in vivo* conditions, fatty acids also interact with other cells, such as macrophages and endothelial cells (162).

## Metabolite Changes During Net Formation

To form NETs, neutrophils release dsDNA as a scaffold decorated with toxic granule proteins (8, 9, 17, 19, 169). Currently, several groups have studied the metabolic requirements of NET formation (9, 43, 58). NET formation requires glycolytic ATP production in order to rearrange cytoskeleton for catapult-like release of cytoplasmic granules together with mitochondrial DNA (8, 9, 17, 62, 169). It has been reported that glycolytic ATP production aids the microtubule network assembly to form NETs (9, 169). Using genetic and pharmacologic approaches, it has been demonstrated that lack of Opa1 reduces the mitochondrial ETC I activity in mouse neutrophils. As a result, the ATP production *via* glycolysis was reduced due to lowered level of NAD^+^ availability. Moreover, we demonstrated that mice lacking Opa1 in neutrophil populations (*Opa1* ^NΔ^) exhibited a less antibacterial defense capability, providing direct evidence for the role of mitochondria in NET formation (9).

NET formation has been suggested to depend on glucose, but not on glutamine (43). It was reported that glucose uptake increased upon phorbol myristate acetate (PMA) to induce NETs (43). Moreover, NET formation depends on increased intracellular ROS production (19, 40, 170, 171). Patients exhibiting chronic granulomatous disease (CGD) suffer from an increased susceptibility to bacterial and fungal infections. The genetic disorder is characterized by defects of the NADPH oxidase which results in impaired ROS production (19, 172). The lack of sufficient ROS production leads subsequently to defects in cytoskeleton reorganization, degranulation, DNA release and ultimately in reduced formation of NETs (8). However, under certain circumstances in absence of NADPH-oxidase activity mitochondria could provide sufficient mitochondrial ROS (mitoROS) as a compensatory mechanism to generate NETs (24). However, the increase in ROS production occurs much faster and seems not to be energy-dependent (43). Additionally, 2-deoxy-d-glucose (2-DG), an inhibitor of glycolysis, can block NET formation (9, 43) by inhibiting glycolysis and PPP.

The NADPH used by NOX originates from the PPP, which demonstrates the importance of a tightly regulated glucose metabolism in neutrophil functions. Hyperglycemia has been demonstrated to enhance the production of NETs (88, 134), which might explain the higher incidence of spontaneous NETs seen in type 2 diabetes patients (88). For example, in high glucose concentration, neutrophils were spontaneously activated, but had less ability to respond to bacterial antigen stimulation such as lipopolysaccharide (LPS) (134). On the other hand, hyperglycemia was also shown to reduce key functions of neutrophils, such as phagocytosis, ROS production and bacterial killing (134, 173), perhaps owing to unstable NETs which in addition contain decreased amounts of anti-microbial peptides compared to NETs released in the presence of physiological glucose concentrations (174, 175).

Withdrawal of glucose from medium leads to complete eradication of NETs (43, 58, 64). When glucose is replenished, the formation of NETs is rescued (58). Upon neutrophil activation, GLUT1 expression is increased on the cell surface of neutrophils in order to uptake glucose for glycolysis (43). Moreover, increases in the extracellular acidification rate and lactate dehydrogenase (LDH) activity were also reported (176), supporting the notion that glycolysis is important in the process of NET formation. Similarly, 6-aminonicotinamide [6-AN, a glucose-6-phosphate dehydrogenase (G6PDH) inhibitor] can disrupt PPP metabolism, which leads to NET formation (58). Subsequently, a metabolic flip toward PPP occurs during NET formation (3, 58), accompanied by increased G6PD activity, which diverts the glycolytic intermediate G6P. In PMA and amyloid fibrils triggered neutrophils, the G6PD inhibitor 6-aminonicotinamide (6-AN) blocked ROS generation and dsDNA release, suggesting that G6P is a fuel for NOX2 activation (58). However, the inhibition of ROS and NETs was incomplete, indicating that there might be additional sources of energy such as an active glycolytic pathway (3, 43, 176).

Furthermore, TKT, a thiamine pyrophosphate (vitamin B1)-dependent enzyme that connects the pentose phosphate pathway and the glycolytic pathway, was shown to be important for the NADPH oxidase activity and consequently for the NET formation. Pharmacological suppression of TKT or a genetic deficit in transaldolase 1, non-oxidative PPP enzymes that aid in the conversion of oxPPP reaction products back into glucose 6-phosphate, reduce oxidative burst and NET formation (177).

**Figure 6:**
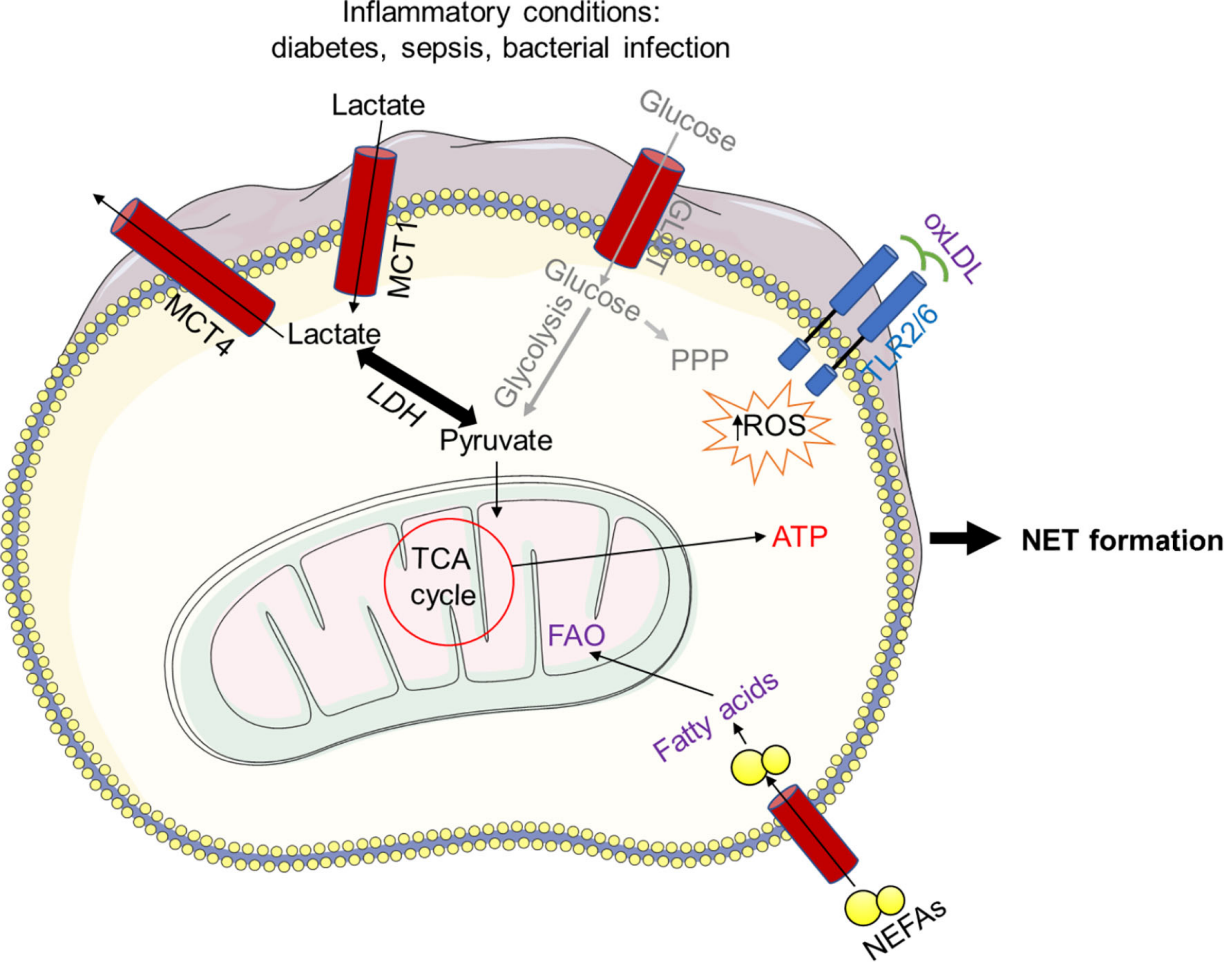
Bacterial lactic acid generated during infection can induce NET formation. Bacteria-produced lactic acid can cause NET formation in bovine neutrophils, and inhibiting the lactate transporter, the mono-carboxylate or lactate dehydrogenase (LDH) enzyme, prevents this. Because LDH can convert both pyruvate and lactate, it is possible that neutrophils use lactate during NET formation and glycolysis to circumvent the PPP. Furthermore, NET formation in human neutrophils is caused by oxidized low-density lipoprotein (oxLDL) in a ROS-dependent manner upon TLR activation. In addition, non-esterified fatty acids (NEFAs), such as oleic acid (OA) and linoleic acid (LA), initiate FAO, allowing increased ATP production and consequently NET formation.

Although neutrophils are regularly found under hypoxic inflammatory conditions in tissues with enriched lactate, the role of lactate in neutrophil activity has received so far little attention only. Given the current paradigm shift in the role of lactate in metabolism, it has recently been reported that lactate plays a unique role in NET formation. Moreover, human neutrophils exogenously treated with lactate exhibit NET formation, albeit NET formation was dramatically reduced when LDH activity was inhibited (3, 9, 43, 176). In addition, the lactic acid produced by bacteria could also induce NET formation by bovine neutrophils (178), and inhibition of its transporter, the monocarboxylate or LDH enzyme, blocks the lactate-induced NET formation (64, 178). LDH has the property to convert both pyruvate and lactate, suggesting that neutrophils exploiting lactate during NET formation and glycolysis bypass the PPP to act as a major metabolic pathway during NET formation. Therefore, the modulation of lactate level under inflammatory conditions, such as diabetes and sepsis, could control neutrophil activation and NET formation. Additionally, oxidized low-density lipoprotein (oxLDL) upon activation of the TLR pathway cause NET formation in human PMNs (131) (**Figure 6**).

Evidence for an important role of the mitochondrial metabolism has recently been obtained when platelet-activating factor (PAF) triggered mitochondrial hyperpolarization and extracellular ATP release *via* pannexin-1, resulting in NET formation. Interestingly, P2X1 purinergic receptor blockage hindered mitochondrial hyperpolarization and NET formation. Hence, ATP and ATP channel pannexin 1 (Panx1) contribute to NET formation and may represent therapeutic targets (179). It has been further concluded that PAF-induced NET release is dependent on glycolysis, mitochondrial ATP synthesis and purinergic signaling (180). Recent studies have revealed the importance of glutamine in NET formation. For example, lack of glutamine or inhibition of complex V to disrupt the mitochondrial ATPase function partially inhibited NET formation, indicating a glutamine-driven mitochondria-dependent metabolism is also utilized for neutrophils to form NETs (3, 64). Mitochondrial translocation toward the cell surface upon activation (8, 9) and its hyperpolarization to release mitochondrial ROS (180), as well as oxidized mitochondrial DNA (24) point to a direct role of mitochondria in NET formation (169, 181, 182).

NETs could potentially be formed independent of the NOX-NADPH oxidase, depending on the availability of sufficient amounts of mitochondrial ROS (24, 33, 134). Some pathogens increase mitochondrial ROS production (183) that may result in reduced dependency on glucose metabolism.

Oxygen level and the pH seem to have an important role in NOX-independent NET formation as well, as it has been demonstrated that, under acidic conditions, glycolysis and ROS production are reduced and therefore less NETs are detected, indicating that conditions at the site of inflammation can also influence the extent of NET formation (184, 185). The role of the metabolism of fatty acids on NET formation is less studied, although it has been reported that non-esterified fatty acids (NEFAs) such as oleic acid (OA) and linoleic acid (LA), that are often associated with infectious diseases, are associated with a higher incidence of infections and NEFAs induced the formation of NETs through extracellular release of ATP *via* Pannexin 1 and subsequent activation of purinergic receptors. Specific blocking of P2X1, a non-selective cation channel, eliminated NET formation upon NEFAs exposure (186) (**Figure 6**). Moreover, other fatty acids, such as Furanoid F-Acid F6, can also activate neutrophils to induce ROS *via* both NADPH oxidase (NOX) and mitochondrial activity, resulting in rapid NET formation. Moreover, Furanoid F-Acid F6 also required protein kinase B (AKT) to induce NET formation (187). Common long-chain fatty acids, such as palmitic acid (PA), palmitoleic acid (PO), stearic acid (SA), and oleic acid caused NET formation *via* NOX, ERK (extracellular signal-regulated kinase) and JNK (c-Jun N-terminal kinase)-dependent pathways. Others reports suggested that chlorinated lipids, 2-chlorofatty aldehyde and 2-chlorofatty acid (2-ClFA), which is the byproduct of MPO release in the phagosome during neutrophil activation, can convert hydrogen peroxide and chloride to hypochlorous acid (HOCl), leading to NET formation (188). This study also demonstrated that human neutrophils exposed to physiological levels of 2-ClFAs induced functional NETs, albeit in the absence of degranulation, and reduced *Escherichia coli* colony forming units. NETs formed by 2-ClFA were calcium and protein arginine deiminase 4 (PAD4)-dependent (188). NET formation can also be induced by oxidized lipoprotein in correlation with ROS production (131), which is supported by the tumor infiltrating c-kit^+^ neutrophils that utilize mitochondrial FAO (91). Important role of diverse metabolic pathways on NET formation have been intensely studied and summarized in **Table 1**.

## Metabolic Pathways in Net-Related Diseases

### Diabetes

Diabetes is a systemic metabolic disorder that is characterized by increased glucose levels (3). Several studies have described impaired neutrophil functions in diabetic patients and animal models, including neutrophil adhesion, chemotaxis, phagocytosis, ROS production, microbicidal activity, mitochondrial potential and ATP levels (66, 189, 190). Hyperglycemia results in decreased G6PD and glutaminase enzymatic activities (66). Consequently, putative compensatory FAO utilization is observed in neutrophils during hyperglycemia due to defective glucose and glutamine metabolism (66). High glucose levels found in diabetic patients are reported to increase NET formation *in vitro* and *in vivo* in association with increased NADPH oxidase activity (84, 86, 88), demonstrating the role of NETs in the pathogenesis of diabetes and its complications like diabetic retinopathy (DR) (83, 87). The development of high glucose-induced NETs was found to be affiliated with the NADPH oxidase machinery. Furthermore, long-term hyperglycemia allows peripheral neutrophils to cross the blood–retinal barrier (BRB) form NETs in the vitreous body and retina. Monitoring NET circulating products in diabetic patients’ serum might be used to assess the severity of DR in order to minimize disease progression and offer prompt therapy (88). Additionally, improved neutrophils phagocytosis and ROS production is reported after treatment of insulin, a common blood glucose lowering therapy (66). Furthermore, GLUT4 is translocated to the cell surface upon activation with PMA in an insulin-sensitive manner and may play a role in the redistribution of glucose during hypoglycemia. In contrast, GLUT1 and GLUT3 are expressed on the surface independent of neutrophil activation and insulin levels (135) (**Table 1**).

### Obesity

Obesity is associated with a risk of higher mortality due to low-grade chronic inflammation (90). Moreover, enhanced neutrophil activity, increased superoxide radicals and NET formation are observed in association with obesity (89, 191). Weight loss of obese patients results in decrease of NET formation (192). While normal neutrophils utilize glycolysis and PPP under both physiological and inflammatory conditions, neutrophils from obese mice exhibit diminished NET release, perhaps owing to “exhaustion” (90). Moreover, the ability to form NETs under *in vivo* condition was reduced in obese mice. Consequently, reduced NET formation and suppressed overactivation of neutrophils due to obesity may promote infections and increase the severity of sepsis (90). Furthermore, GLUT1 surface expression was reduced in LPS-activated neutrophils of high-fat diet mice, while GLUT1 expression was unaffected in resting neutrophils of these mice (90) (**Table 1**).

### Cystic Fibrosis

Cystic Fibrosis (CF) is a genetic syndrome characterized by mutations in the CF transmembrane conductance regulator gene (193). High levels of free glucose and amino acids are found in CF airway lumen of CF patients (122). CF neutrophils exhibit suppressed apoptosis compared to normal neutrophils (193). Furthermore, an increased capacity of NET formation is observed in CF neutrophils (123), a phenomenon, which has been linked with impairment of the obstructive lung function (120). CF airway neutrophils, but not blood neutrophils, from CF patients exhibit increased expression of GLUT1, neutral amino acids transporter 2 (ASCT2) and inorganic phosphate transporter 1 & 2 (PiT1, PiT2) compared to normal neutrophils, suggesting that metabolic adaption processes occur in neutrophils upon their recruitment from blood into CF airway lumen (122). ROS production is decreased in airway CF neutrophils in contrast to blood neutrophils (121). Furthermore, GLUT1 and PiT2 levels in blood neutrophils are diminished upon oral treatment with corticosteroids (122) (**Table 1**).

### Cancer

Tumor-associated neutrophils (TANs) can exhibit both pro- and antitumor properties (3). In mouse lung adenocarcinoma, TANs display increased GLUT1 expression and glucose metabolism. These findings have been associated with a decelerated neutrophil turnover in tumors, increased tumor growth, and diminished efficacy of radiotherapy (94). Immature low-density neutrophils (iLDNs) rely on mitochondria-dependent ATP production through the catabolism of glutamate and proline. It has been shown that they can exhibit a pro-metastatic function under nutrient-deprived conditions (92). Additionally, neutrophils are able to adapt an oxidative phenotype through c-KIT signaling in response to tumor. Subsequent ROS production by NADPH oxidase allows ROS-mediated suppression of T cells even in glucose-limited environment and favors tumor growth (91). Furthermore, neutrophils are attracted by tumor growth to infiltrate and form NETs that modulate the metabolic profile of the tumors leading to its increased survival (93). NETs formed from TANs and their associated proteins elastase and matrixmetalloproteinase-9 also facilitate the reestablishment of the proliferative capacity of dormant lung cancer cells through extracellular matrix remodeling (95). Moreover, NETs are reported to trap circulating tumor cells leading to increased metastatic formation (194). Neutrophil infiltration in human thyroid cancer (TC) has been recently reported to correlate with tumor growth. Moreover, TC-derived conditioned media (CM) attracted neutrophils, increased their survival, caused morphological alterations in neutrophils, and triggered and altered neutrophil kinetic characteristics. Interestingly, anaplastic (ATC) CM-primed neutrophils produced NETs containing mitochondrial DNA in a mitochondrial ROS-dependent and cell death-independent manner (26, 195) (**Table 1**).

### Systemic Lupus Erythematosus

Systemic lupus erythematosus (SLE) is an autoimmune disease characterized by a high degree of immune inflammation and self-destruction, as well as defective clearance of NETs (97, 101). Neutrophils of SLE patients exhibit dysregulated aggregation, phagocytosis, oxidative burst and increased NET formation (3). Deficient GLUT3 and GLUT6 expression in SLE neutrophils results in increased intracellular basal lactate production and decreased ATP production. The decreased expression of GLUT3 and GLUT6 results in a decreased ability to uptake glucose, resulting in decreased glycolytic activity (100). Furthermore, intracellular glutathione (GSH) levels and γ-glutamyl-transpeptidase activity are reduced in neutrophils of patients with active SLE leading to reduced redox capacity (100). NETs are induced by ribonucleoprotein immune complexes (RNP-ICs) found in SLE through augmented production of mitochondrial ROS production (24). Low-density granulocytes (LDGs), a distinct subset of pro-inflammatory neutrophils, have also been described in SLE patients (24), and are associated with enhanced disease activity (196). LDGs exhibit increased mitochondrial ROS (24) associated with increased NET formation (99). Furthermore, NET formation by LDGs is shown to be responsible for endothelial damage (96, 98). Lupus symptoms are rescued through treatment with 2-DG, metformin and rapamycin in mice models, confirming a potential role of metabolic alterations in SLE (197) (**Table 1**). It remains unclear why NET formation is increased in SLE neutrophils in spite of reduced expression of glucose transporters on these cells. Perhaps, other factors, such as increased mitochondrial ROS activity, can compensate reduced glycolytic activity.

### Rheumatoid Arthritis

Rheumatoid arthritis (RA) is an autoimmune disease characterized by synovitis and joint destruction through extensive activation of the immune system (3). Infiltrated neutrophils in joint synovial fluid (SF) release cytotoxic proteases (198) and show high ROS production (198) and increased expression of cytokines (103, 199). The hypoxic conditions in joints of RA patients increase the survival of SF neutrophils (103). Impaired beta cell function and insulin resistance in RA patients leads to a higher risk for diabetes (200). Interestingly, enhanced and accelerated NET formation is observed in RA patients (104). In RA, the NETs are involved in the degradation of cartilage as well as in the activation of fibroblast-like synoviocytes and macrophages, resulting ultimately in joint damage (106). Within RA joint milieu, due to low oxygen availability, HIF-1α is upregulated in neutrophils leading to increased glycolytic activity by upregulating the expression of G3PDH and triosephosphate isomerse-1 to provide ATP energy supply (102) (**Table 1**).

### Atherosclerosis

Plaques containing LDL and cholesterol, as well as hyperlipidemia and inflammation, are the hallmarks of atherosclerosis (3). Neutrophil functions modulated by metabolic changes are reported to play a role in the pathogenesis of atherosclerosis (201, 202). Accordingly, hyperlipidemia and hypercholesterolemia trigger atherosclerosis through induction of neutrophilia (201). NET formation is induced by oxidized LDL and cholesterol crystals (131). Furthermore, the decrease of CXCL1 levels, resulting in inhibition of NAMPT and NAD^+^ biosynthesis, leads to diminished neutrophil infiltration in atherosclerotic plaques (203). NETs were also seen in atherosclerotic lesions in mice, together with an elevation in the pro-inflammatory markers IL-1 and IL-6 (204). Moreover, luminally adherent neutrophils releasing DNA in apolipoprotein-deficient (*Apoe* ^-/-^) mice fed a high-fat diet for 4–6 weeks were identified using a two-photon microscopy intravital technique, but no neutrophil adhesion and thus no NET release were observed in mice receiving chow diet (130, 132, 182) (**Table 1**).

### COVID-19

COVID-19 is a proinflammatory condition with a disproportionately high incidence of developing acute respiratory distress syndrome (ARDS), acute renal failure, arrhythmia, and shock (205, 206). A number of NET-associated indicators, such as cell-free DNA (cfDNA), MPO/NE-DNA complexes, and citrullinated histone H3 (CitH3) are considerably elevated in the circulation and/or tracheal aspirates of COVID-19 patients (127, 129, 207–215). *In vitro* treatment of neutrophils isolated from healthy controls with serum/plasma from COVID-19 patients led to formation of extracellular DNA traps, confirming the presence of NET-inducing substances in the circulation of COVID-19 patients (127, 129, 215–218). Furthermore, neutrophils obtained from COVID-19 patients also exhibited increased spontaneous NET formation *in vitro* (127, 207, 209, 210).

Several metabolic pathways were reported to be dysregulated in neutrophils from COVID-19 patients such as the tryptophan metabolism, TCA cycle, polyunsaturated fatty acid mobilization, and eicosanoid biosynthesis. In addition to these metabolic changes, it has also been reported that COVID-19 viral infection itself can cause metabolic reprograming in both neutrophils and PBMC (219, 220). Recent studies confirmed elevated levels of inflammatory cytokines, such as IL-1β and IL-6, as well as insufficient IL-10 responses in individuals with severe COVID-19. Furthermore, soluble tumor necrosis factor receptor 1 (sTNFR1), a surrogate receptor for circulating TNF-α, and pyruvate kinase M2 (PKM2) were significantly increased as well in COVID-19 patients. PKM2, an enzyme that catalyze the final step of glycolysis is a coactivator of HIF-1α (221), which induces the expression of proglycolytic enzymes, such as LDH, PDKs (pyruvate dehydrogenase kinases), and GLUT1. Additionally, enhanced cytosolic lactate and succinate levels have been detected in COVID-19 neutrophils compared to healthy control neutrophils.

Interestingly, accumulation of cytosolic succinate resulted in decreased breakdown of HIF-1α (222) and therefore enhanced the cytosolic levels of HIF-1α in COVID-19 neutrophils (124). The increased cytosolic lactate: pyruvate ratio has been revealed as fundamental metabolic change, suggesting a shift towards glycolysis in COVID-19 neutrophils (124). Glycolysis and lactate production are crucial processes in the development of neutrophil antimicrobial responses (43, 176). The increased glycolytic activity of COVID-19 neutrophils might provide a mechanistic explanation for the increased spontaneous NET formation by neutrophils isolated from patients with severe COVID-19 (209, 210). However, contrasting results have also been reported that suggested an impaired glycolytic flow and oxidative metabolism in neutrophils isolated from COVID-19 patients with ARDS symptoms (223). Currently, the reasons for these different findings remain unclear.

### Sepsis

Sepsis is a systemic inflammatory syndrome characterized by impaired neutrophil migration and functions (3). Reduced expression of chemokine receptor CXCR2, GPCR receptor kinase and impaired actin polymerization cause defective neutrophil chemotaxis in sepsis (224, 225). Furthermore, impaired mitochondrial activity, largely complex V (ATP synthesis) and ETC complex III and IV were linked to dysfunction of neutrophil chemotaxis driven by LPS in septic conditions (113). Increased autophagy in association with augmented NET formation has been reported to improve the survival of sepsis patients (116). The induction of autophagy during sepsis leads to functional rejuvenation of neutrophil population and alteration of neutrophil metabolism through production of intermediate products (116). On the other hand, enhanced NET levels are associated with increased sepsis severity and subsequent organ damage (111, 117). NETs are accompanied with intestinal barrier dysfunction, ER stress activation and ROS production in sepsis patients, leading to increased serum intestinal fatty-acid binding protein (I-FABP) and D-lactate that positively correlates with NET formation, indicating that fatty acid and lipid metabolism might be required for neutrophil maturation during sepsis (**Figure 5**) (63, 119). Moreover, peroxisomal lipid synthesis regulated inflammation by sustaining neutrophil membrane phospholipid composition and viability (110). Increased expression of G6PD and phosphogluconate dehydrogenase suggest an induced PPP pathway as well (**Figure 2**) (114, 118). Additionally, plasma ATP is required for neutrophil activation during sepsis (226), while systemic ATP reduces neutrophil activation and chemotaxis through the disruption of endogenous purinergic signaling mechanisms (115). Accordingly, blockage of endogenous ATP signaling by suramin, a P2-receptor antagonist, increases bacterial growth and mortality whereas apyrase, an ATP diphosphatase, improves survival in sepsis through improved bacterial clearance and removal of systemic ATP (115) (**Table 1**).

### Glucose-6-Phosphate Dehydrogenase (G6PD) Deficiency

G6PD deficiency is an inherited enzymatic disorder characterized by a decrease in NADPH production, resulting in glutathione depletion and hemolytic anemia (3, 227, 228). G6PD is required for the switch of the cellular metabolism from glycolysis to PPP (229). Moreover, G6PD activity is increased after PMA stimulation in neutrophils and switches the metabolism toward the PPP pathway, resulting in increased NET formation. On the other hand, in the absence of G6PD activity, the PPP metabolism and NET formation is diminished (58). Neutrophils exhibiting G6PD deficiency, similar to mild CGD, showed impaired microbicidal and metabolic activity (107). Typically, bactericidal activity and neutrophil function are not impaired in mild G6PD-deficient neutrophils (108, 109). However, in patients with severe enzymatic deficiency reduced G6PD activity, increased susceptibility to infections due to reduced NADPH generation and subsequently impaired NET formation have been observed (109). Interestingly, rapid neutrophil turnover and diurnal fluctuating levels of G6PD activity are sufficient to induce an effective respiratory burst (230). Furthermore, nicotinamide nucleotide biosynthesis is suggested to be sufficient to compensate the G6PD deficiency, resulting in comparable rates of NET formation in G6PD patients and healthy controls (231) (**Table 1**).

## Conclusions

Neutrophils are capable of adapting to the tissue environment under pathological conditions by modifying their metabolic activity utilizing several metabolic pathways. An increased mitochondrial activity and its contribution within a network of neutrophil metabolic pathways has been shown to be critical for anti-bacterial functions mediated by neutrophils. Pathological changes of these pathways contribute to disease, resulting in either immunodeficiency or accelerated inflammatory responses. The molecular mechanism of NET formation heavily relies on an intact cellular metabolism in neutrophils.

## Author Contributions

Conceptualization: DS, SY, and H-US. Methodology: DS, LG, and SP. Investigation: DS, LG, and SP. Visualization: DS, LG, SP, and SY. Funding acquisition: SY and H-US. Project administration: DS, SY, and H-US. Supervision: SY and H-US. Writing – original draft: DS, SY, and H-US. Writing – review & editing: DS, RL, PR, AK, AR, NB, SY, and H-US. All authors contributed to the article and approved the submitted version.

## Funding

Swiss National Science Foundation, grant No. 31003A_173215 (SY); Swiss National Science Foundation, grant No. 310030_184816 (H-US); Russian Government Program “Recruitment of the Leading Scientists into the Russian Institutions of Higher Education”, grant No. 075-15-2021-600 (H-US). AR was partially supported by Kazan Federal University (KFU) Strategic Academic Leadership Program.

## Conflict of Interest

The authors declare that the research was conducted in the absence of any commercial or financial relationships that could be construed as a potential conflict of interest.

## Publisher’s Note

All claims expressed in this article are solely those of the authors and do not necessarily represent those of their affiliated organizations, or those of the publisher, the editors and the reviewers. Any product that may be evaluated in this article, or claim that may be made by its manufacturer, is not guaranteed or endorsed by the publisher.

## Glossary

**Table:**

2-ClFA	2-chlorofatty aldehyde and 2-chlorofatty acid
2-DG	2-deoxy-d-glucose
6-AN	6-aminonicotinamide
PGD	6-phosphogluconate dehydrogenase
acetyl-CoA	acetyl-coenzyme A
ATP	adenosine triphosphate
ASCT2	amino acids transporter 2
ATC	anaplastic thyroid cancer
AMPs	antimicrobial peptides
Apoe-/-	apolipoprotein-deficient
Panx1	ATP channel pannexin 1
JNK	c-Jun N-terminal kinase
CPT-1	carnitine palmitoyltransferase-1
CACT	carnitine-acylcarnitine translocase
CPT-2	carnitine palmitoyltransferase-2
cfDNA	cell-free DNA
CGD	chronic granulomatous disease
CitH3	citrullinated histone H3
CM	conditioned media
COVID-19	coronavirus disease-2019
CF	cystic fibrosis
DR	diabetic retinopathy
FADH2	dihydroflavine-adenine dinucleotide
ETC	electron transport chain
ER	endoplasmic reticulum
ECAR	extracellular acidification rate
ERK	extracellular signal-regulated kinase
FAO	fatty acid oxidation
FAS	fatty acid synthesis
F6P	fructose-6-phosphate
G6P	glucose-6-phosphate
G6Pase-β	glucose-6-phosphatases- β
GLUT	glucose transporter
G6PT	glucose-6-phosphate transporter
G6PD	glucose-6-phosphate dehydrogenase
Grx1	glutaredoxin 1
GSD-1β	glycogen storage disease type Iβ
G3P	glyceraldehyde-3-phosphat
HMP	hexose monophosphate shunt
HK2	hexokinase 2
HOCl	hypochlorous acid
HIF-1α	hypoxia-inducible factor-1α
IL	interleukin
LPS	lipopolysaccharide
LDH	lactose dehydrogenase
LA	linoleic acid
LDGs	low-density granulocytes
LDL	low-density lipoprotein
iLDNs	immature low-density neutrophils
MPO	myeloperoxidase
mtDNA	mitochondrial DNA
∆Ψm	mitochondrial potential
mitoROS	mitochondrial ROS
NETs	neutrophil extracellular traps
NADPH	nicotinamide adenine dinucleotide phosphate
NAD+	nicotinamide adenine dinucleotide
NADP+	nicotinamide-adenine dinucleotide phosphate
NAMPT	nicotinamide phosphor-ribosyl transferase
NO	nitric oxide
NEFAs	non-esterified fatty acids
OA	oleic acid
OPA1	optic atrophy 1
OCR	oxygen consumption rate
OXPHOS	oxidative phosphorylation
PA	palmitic acid
PO	palmitoleic acid
PPP	pentose-phosphate pathway
PBMCs	peripheral blood mononuclear cells
PMA	phorbol-12 myristate 13-acetate
PiT1, PiT2	phosphate transporter 1 & 2
PFK	phosphofructokinase
PAF	platelet-activating factor
PAD4	protein arginine deiminase 4
PDK	pyruvate dehydrogenase kinase
PDH	pyruvate dehydrogenase
PKM2	pyruvate kinase M2
ROS	reactive oxygen species
ARDS	respiratory distress syndrome
R5P	ribose-5-phosphate
RNP-ICs	ribonucleoprotein immune complexes
RA	rheumatoid arthritis
SGLT2	sodium glucose cotransporter 2
SNAT	sodium-coupled neutral amino acid transporter
SLCs	solute carrier-type transporters
sTNFR1	soluble tumor necrosis factor receptor 1
TNF-α	tumor necrosis factor alfa
SA	stearic acid
SF	synovial fluid
SLE	systemic lupus erythematosus
TCA	tricarboxylic acid cycle
TKT	transketolase
TANs	tumor-associated neutrophils
TC	thyroid cancer
